# Draft Genome Sequences of 26 *Porphyromonas* Strains Isolated from the Canine Oral Microbiome

**DOI:** 10.1128/genomeA.00187-15

**Published:** 2015-04-09

**Authors:** David A. Coil, Alexandra Alexiev, Corrin Wallis, Ciaran O’Flynn, Oliver Deusch, Ian Davis, Alexander Horsfall, Nicola Kirkwood, Guillaume Jospin, Jonathan A. Eisen, Stephen Harris, Aaron E. Darling

**Affiliations:** a University of California Davis Genome Center, Davis, California, USA; b The Waltham Centre for Pet Nutrition, Melton Mowbray, Leicestershire, United Kingdom; c Department of Evolution and Ecology and Department of Medical Microbiology and Immunology, University of California Davis, Davis, California, USA; d ithree institute, University of Technology, Sydney, Australia

## Abstract

We present the draft genome sequences for 26 strains of *Porphyromonas* (*P. canoris*, *P. gulae*, *P. cangingavalis*, *P. macacae*, and 7 unidentified) and an unidentified member of the *Porphyromonadaceae* family. All of these strains were isolated from the canine oral cavity, from dogs with and without early periodontal disease.

## GENOME ANNOUNCEMENT

Members of the *Porphyromonas* genus have been shown to be present in periodontal disease in both humans and dogs (1, 2). As part of a larger study on canine periodontal disease (1), a number of *Porphyromonas* strains were isolated, cultured, and sequenced.

The study cohort comprised client-owned pet dogs that presented at a veterinary referral dental clinic (The Veterinary Dental Surgery, Surrey, United Kingdom). Only dogs under anesthetic for routine dental treatment or other noninfectious conditions were included in the study. No dogs were anesthetized solely for the collection of plaque samples. For further details see our previously published work on this cohort (1).

Bacterial isolates were grown on Columbia blood agar (CBA) containing 5% defibrinated horse blood supplemented with 5 mg/L Hemin (catalog no. H9039; Sigma) and 0.5 mg/L Menadione (catalog no. M5625; Sigma). The isolates were incubated at 38°C in an anaerobic cabinet (DonWhitley Scientific Ltd., Shipley, United Kingdom) (80% nitrogen, 10% hydrogen, and 10% carbon dioxide) for 1 to 21 days. DNA extraction was performed on scrapings resuspended in 3 mL brain heart infusion broth (BHI) (catalog no. CM1135; Oxoid), using the Joint Genome Institute DNA isolation bacterial cetyltrimethylammonium bromide (CTAB) protocol.

Following genomic DNA extraction, 16S rRNA genes were amplified by PCR using 16S universal primers. Two forward primers, AC84 (5′ AGA GTT TGA TYM TGG CTC AG 3′) and AC83 (5′ AGG GTT CGA TTC TGG CTC AG 3′, which contains sequence specific to the *Bifidobacteriaceae*), were used. Both primers are homologous to *Escherichia coli* position 8 to 27. The reverse primer was C72 (5′ GYT ACC TTG TTA CGA CTT 3′), which is homologous to *E. coli* position 1492 to 1509.

Two Illumina sequencing library preparation protocols were used, one based on mechanical shearing of DNA, and another based on tagmentation. The tagmentation libraries were constructed using the Nextera DNA sample prep kit (Epicentre) according to the manufacturer’s instructions. The libraries were size selected (300 to 600 nucleotides) on a PippinPrep instrument (Sage Science). For the mechanical shearing libraries, genomic DNA was subjected to sonication using a Bioruptor sonication device (Diagenode) programmed to generate 200 to 300 nucleotide fragments. These fragments were then transferred to an automated DNA library preparation platform Apollo 324 (IntegenX), where steps of end-repair, A-tailing, and bar code adapter ligation were carried out. Subsequently, adapter-ligated samples were subjected individually to 11 cycles of PCR amplification (with a Qiagen kit, [catalog no. 201205]), cleaned up, and size selected (320 bp) on a PippinPrep device (Sage Bioscience). All libraries were sequenced on an Illumina HiSeq 2000 machine.

All sequence processing and assembly of the Illumina reads were performed using the A5 assembly pipeline (3). Automated annotation was performed using the RAST annotation server (4). The assembly and annotation statistics are presented in Table 1.

**Table 1:** Accession numbers and assembly statistics for 26 *Porphyromonas* strains

Strain identifier	Accession no.	No. of contigs	No. of scaffolds	Genome size (bp)	*N*_50_	No. of raw reads	Coverage (×)
*Porphyromonas canoris* COT-108_OH1224	JQZX00000000	93	21	2,308,674	356,707	11,771,344	358
*Porphyromonas* sp. COT-108_OH1349	JRAH00000000	126	43	2,330,607	294,161	6,895,102	194
*Porphyromonas gulae* I COT-052_OH1355	JRAG00000000	151	40	2,340,299	162,065	9,130,698	357
*Porphyromonas cangingivalis* COT-109_OH1379^*a*^	JQJF00000000	58	21	2,364,758	227,673	6,682,444	205
*Porphyromonas cangingivalis* COT-109_OH1386	JQJD00000000	145	66	2,441,844	100,405	6,061,670	149
*Porphyromonas gingivicanis* COT-022_OH1391^*a*^	JQZW00000000	53	20	1,983,669	255,048	8,862,612	329
*Porphyromonas* sp. COT-239_OH1446	JRAO00000000	62	37	1,961,776	133,546	4,575,892	150
*Porphyromonas crevioricanis* COT-253_OH1447	JQJC00000000	63	30	2,161,087	144,469	8,455,142	263
*Porphyromonas gulae* I COT-052_OH1451	JRAI00000000	134	91	2,463,757	46,861	13,491,026	371
*Porphyromonas crevioricanis* COT-253_OH2125	JQJB00000000	76	14	2,105,287	491,012	8,101,504	358
*Porphyromonas gulae* I COT-052_OH2179	JRAJ00000000	165	26	2,442,276	247,543	13,797,658	527
*Porphyromonas macacae* COT-192_OH2631	JRFB00000000	102	54	2,316,420	1,255,122	14,360,788	572
*Porphyromonas canoris* OH2762	JQZV00000000	80	14	2,202,536	351,685	12,612,176	539
*Porphyromonas gulae* II COT-052_OH2857	JRFD00000000	143	53	2,333,470	78,104	15,364,628	597
*Porphyromonas macacae* COT-192_OH2859	JRFA00000000	129	34	2,364,070	207,096	11,085,904	426
*Porphyromonas* sp. COT-108_OH2963	JRAP00000000	53	21	2,179,370	225,693	5,782,088	164
*Porphyromonas gulae* OH3161B	JQJE00000000	161	47	2,335,601	149,377	12,527,276	500
*Porphyromonas gulae* II COT-052_OH3439	JRAK00000000	290	163	2,588,710	55,832	7,989,956	192
*Porphyromonas gulae* I COT-052_OH3471	JRAQ00000000	129	44	2,371,923	134,999	4,743,702	123
*Porphyromonas gulae* II COT-052_OH3498	JRAF00000000	102	71	2,252,877	67,961	8,041,104	312
*Porphyromonas* sp. COT-290_OH3588CRE	JRFC00000000	105	48	2,294,016	183,859	14,279,656	588
*Porphyromonas gulae* II COT-052_OH3856	JRAT00000000	117	31	2,388,773	131,574	14,306,028	557
*Porphyromonas gulae* II COT-052_OH4119	JRAL00000000	143	52	2,287,427	154,457	12,704,688	518
*Porphyromonadaceae* [G-1] sp. COT-184_OH4590	JRAN00000000	191	79	2,392,483	66,072	6,627,810	178
*Porphyromonas* sp. UQD_349_COT-052_OH4946	JQZY00000000	131	34	2,384,876	169,169	10,619,640	414
*Porphyromonas* sp. COT-290_OH860	JRAR00000000	122	82	2,343,073	60,215	7,717,532	206

a These libraries were constructed with mechanical shearing; all others were constructed by tagmentation.

### Nucleotide sequence accession numbers.

All 26 assemblies described in this paper have been deposited as whole-genome shotgun projects in DDBJ/EMBL/GenBank under the accession numbers provided in Table 1.
